# Supplementary material for: Impella versus Venoarterial Extracorporeal Membrane Oxygenation for Acute Myocardial Infarction Cardiogenic Shock: A Systematic Review and Meta-Analysis
Source: J Clin Med. 2022 Jul 7;11(14):3955. doi: 10.3390/jcm11143955 (PMC9317942; doi:10.3390/jcm11143955)
Supplement: Supplementary file 1 [file jcm-11-03955-s001.zip › File S1- MEDLINE Search Strategy.pdf]

Supplement 1: Sample MEDLINE search strategy

1 exp Extracorporeal Membrane Oxygenation/ (12811)
2 (ecmo or vaecmo or va ecmo or extracorporeal membrane oxygenation or extracorporeal life support or ecls).mp. (16540)
3 1 or 2 (16540)
4 exp heart-assist devices/ (15949)
5 (impella* or impella 5* or impella 2* or impella CP).mp. (866)
6 (microaxial* adj4 (pump or ventricular assist or device)).mp. (107)
7 4 or 5 or 6 (16108)
8 3 and 7 (1429)
9 exp myocardial ischemia/ or exp acute coronary syndrome/ or exp myocardial infarction/ (449471)
10 exp myocardial revascularization/ or exp percutaneous coronary intervention/ or exp coronary artery bypass/ (114588)
11 (STEMI or NSTEMI or unstable angina or myocardial infarction or myocardial ischa* or acute coronary syndrome or percutaneous coronary intervention* or angioplast*).mp. (326285)
12 9 or 10 or 11 (553991)
13 exp shock, cardiogenic/ (9554)
14 ((cardiogenic or circulat*) adj4 (shock or failure)).mp. (19701)
15 ((low* or decreas* or declin* or reduc*) adj2 (card* or heart) adj output*).mp. (7805)
16 exp Cardiac Output, Low/ (5558)
17 13 or 14 or 15 or 16 (31612)
18 12 or 17 (570524)
19 8 and 18 (688)
20 exp animals/ not humans.sh. (4911209)
21 19 not 20 (677)
