# Supplementary material for: Impella versus Venoarterial Extracorporeal Membrane Oxygenation for Acute Myocardial Infarction Cardiogenic Shock: A Systematic Review and Meta-Analysis
Source: J Clin Med. 2022 Jul 7;11(14):3955. doi: 10.3390/jcm11143955 (PMC9317942; doi:10.3390/jcm11143955)
Supplement: Supplementary file 1 [file jcm-11-03955-s001.zip › File S2 - Risk of Bias Analysis.pdf]

Supplement 2: Risk of bias analysis

Reference (year)	Selection (max 4)				Comparability (max 2)	Intervention/Exposure (max 3)			Total (max 9)
	Representative of exposed cohort	Selection of non-exposed cohort	Ascertainment of exposure	Outcome not at start	Comparability	Assessment of outcome	Length of follow-up	Adequacy of follow-up	
Mourad (2018)	1	1	1	0	1	1	1	1	7
Garan (2019)	1	1	1	0	0	1	1	1	6
Karami (2020)	1	1	1	0	2	1	1	1	8
Lemor (2020)	1	1	1	0	2	1	1	1	8
Karatolios (2021)	1	1	1	0	2	1	1	1	8
Syntila (2021)	1	1	1	0	2	1	1	1	8
