# Supplementary material for: Impella versus Venoarterial Extracorporeal Membrane Oxygenation for Acute Myocardial Infarction Cardiogenic Shock: A Systematic Review and Meta-Analysis
Source: J Clin Med. 2022 Jul 7;11(14):3955. doi: 10.3390/jcm11143955 (PMC9317942; doi:10.3390/jcm11143955)
Supplement: Supplementary file 1 [file jcm-11-03955-s001.zip › File S4 - Subgroup and Sensitivity Analyses.pdf]

Supplement 4: Subgroup and sensitivity analysis

Subgroup	Mortality	Studies	Pooled Effect Estimate (95% CI)	I^2 statistic
Low risk of bias	In-hospital	4	0.89 (0.82-0.96)	0%
	6-12 months	3	0.87 (0.77-0.98)	0%
Propensity-matched cohorts	In-hospital	3	0.72 (0.59-0.86)	35%
	6-12 months	2	0.88 (0.72-1.09)	0%
No population overlap	In-hospital	4 (minus Karatolios)	0.91 (0.83-0.99)	0%
	6-12 months	4 (minus Karatolios)	0.88 (0.74-1.04)	0%
No population overlap	In-hospital	4 (minus Syntila)	0.90 (0.83-0.98)	0%
	6-12 months	4 (minus Syntila)	0.86 (0.75-0.99)	0%

CI, confidence interval.
