# Supplementary figures and images for: Impella versus Venoarterial Extracorporeal Membrane Oxygenation for Acute Myocardial Infarction Cardiogenic Shock: A Systematic Review and Meta-Analysis
Source: J Clin Med. 2022 Jul 7;11(14):3955. doi: 10.3390/jcm11143955 (PMC9317942; doi:10.3390/jcm11143955)

### Supplement 3: Publication Bias (by in-hospital mortality as outcome)

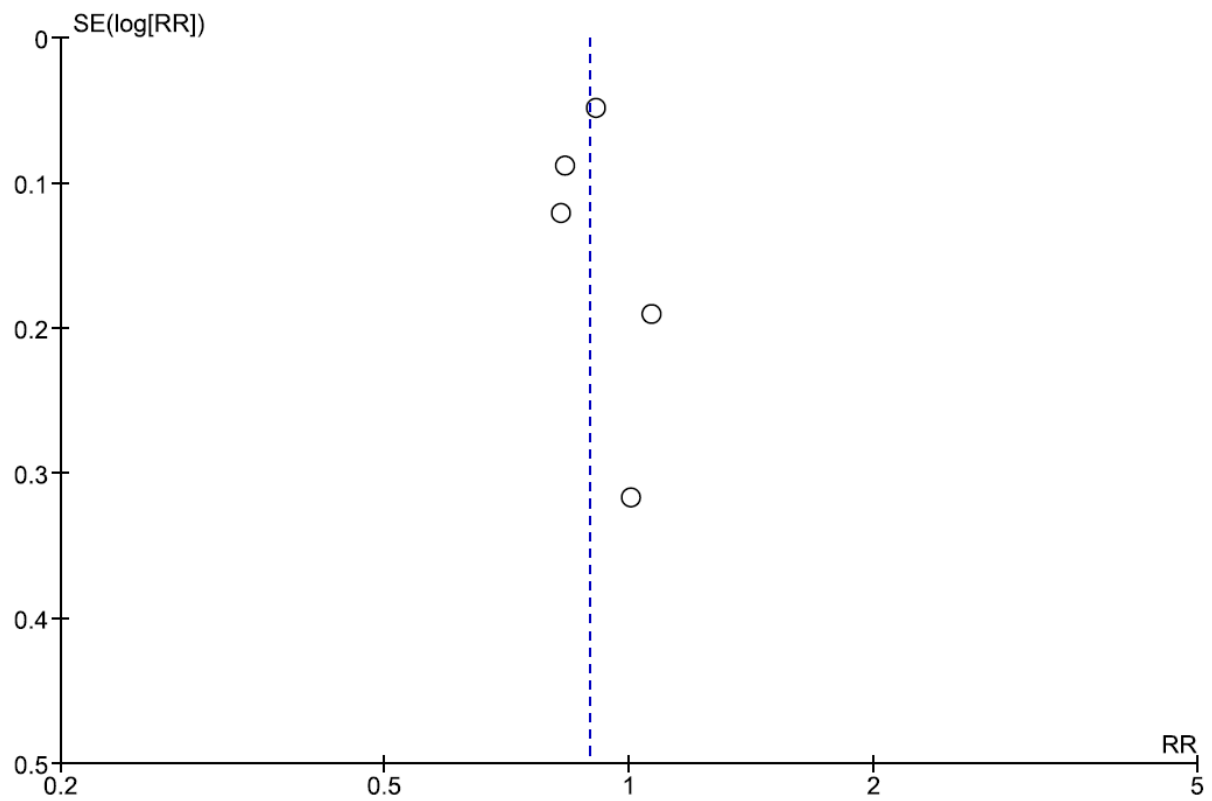

Supplement: Supplementary file 1 [file jcm-11-03955-s001.zip › File S3 - Funnel Plots.pdf]
